# CDK5-mediated phosphorylation and stabilization of TPX2 promotes hepatocellular tumorigenesis

**DOI:** 10.1186/s13046-019-1297-6

**Published:** 2019-07-04

**Authors:** Fuqiang Wang, Wenxing Zhao, Yuehong Gao, Jiechao Zhou, Huifang Li, Guanyun Zhang, Dong Guo, Chengrong Xie, Jie Li, Zhenyu Yin, Jie Zhang

**Affiliations:** 1 Department of Hepatobiliary Surgery, Zhongshan Hospital of Xiamen University, Fujian Provincial Key Laboratory of Chronic Liver Disease and Hepatocellular Carcinoma, Xiamen, 361004 Fujian China; 2 Taian City Central Hospital, Taian, 271000 Shandong China; 3 Fujian Provincial Key Laboratory of Neurodegenerative Diseaseand Aging Research, Institute of Neuroscience, School of Medicine, Xiamen University, Xiamen, 361102 Fujian China; 4 Solomon H. Snyder Department of Neuroscience, Johns Hopkins University School of Medicine, Baltimore, MD 21205 USA

**Keywords:** Cyclin-dependent kinase 5, Hepatocellular carcinoma, Target protein for Xklp2, Tamoxifen

## Abstract

**Background:**

CDK5, an atypical member of the CDK family, play a significant role in the tumorigenesis of multiple organ, but CDK5 and its substrates in genesis and development of HCC is still unclear.

**Methods:**

Expression of CDK5 in HCC tumor and paired adjacent noncancerous tissues from 90 patients were measured by Western blotting, immunohistochemistry, and real-time PCR. The role of CDK5 in cell function and tumorigenesis was explored in HCC cell lines, ex vivo xenografts and diethylnitrosamine induced HCC model. Furthermore, comparative phosphoproteomic screening identified the oncoprotein TPX2 as a new substrate of CDK5. We also identified the effect of CDK5/P25 interaction blocker tamoxifen on HCC cell growth and migration.

**Results:**

CDK5 was increased in HCC tisues and the level of CDK5 was correlated with the severity of HCC based on patient recurrence and 5-year fatality rate. Exogenously expressed CDK5 but not kinase-dead CDK5 promoted proliferation, migration, and invasion of HCC cells. Functional ablation of CDK5 significantly inhibited the exacerbation of HCC cells. Xenograft implantation of HCC cells overexpressing CDK5 promoted tumorigenesis, and genetic knockdown of CDK5 reduced HCC growth and metastasis in vivo. More importantly, heterozygous knockout CDK5 (Cdk5+/−) attenuated HCC tumorigenesis induced by diethylnitrosamine. CDK5-mediated phosphorylation of TPX2 at serine 486 promoted its protein stability. TPX2 silence could restore HCC cell migration capability with overexpression CDK5. Treatment with tamoxifen inhibited cell growth and migration of HCC, demonstrating the role of active CDK5 in HCC.

**Conclusions:**

Our results suggest activation of CDK5 is associated with HCC tumorigenesis. CDK5-mediated phosphorylation and stabilization of TPX2 promotes hepatocellular proliferation and tumorigenicity.

**Electronic supplementary material:**

The online version of this article (10.1186/s13046-019-1297-6) contains supplementary material, which is available to authorized users.

## Background

Hepatocellular carcinoma (HCC), with more than 700,000 new cases annually, is the third most common cause for cancer death worldwide [1, 2]. Despite the improvement in clinical treatments, such as resection, liver transplantation, and ablation, the mortality rate of patients with HCC remains high. High recurrence and metastases after resection are the main causes. Limited drugs, such as sorafenib, have shown survival benefits for individuals with advanced HCC. However, they only increase survival for several months [3]. Elucidating new mechanisms of HCC progression is vital for developing novel therapeutic targets for advanced HCC.

Abnormal expression and activation of cyclin-dependent kinases (CDKs) contribute to tumorigenesis of certain types of cancers [4, 5]. Among CDK family proteins, CDK5 is a unique member that is activated by non-cyclin protein P35 and P39 [6]. The high neuronal expression of P35 and P39 implies that CDK5 exerts its major function in the central nervous system in regulating brain development and neuronal cell survival [7, 8]. Outside of the brain, CDK5 has been recently found to participate in several pathological conditions, such as cancer, diabetes, inflammation, and senescence [9–12]. Accumulating evidence suggests that CDK5 may also play a significant role in the tumorigenesis. Elevated CDK5 and its kinase activity have been reported in different types of cancers including breast cancer, lung cancer, liver cancer, pancreatic cancer, thyroid carcinoma, and myeloma [9, 13–16]. Our recent finding indicated that CDK5 can also suppress gastric tumorigenesis [17]. Thus, CDK5 plays a significant role in tumor progression and metastasis. For HCC, Vollmar et al. have recently reported that overactivation of CDK5 is involved in HCC progression [16, 18]. However, the downstream substrates of CDK5 during HCC progression, which are important to demonstrate the function of CDK5 and develop therapeutic drugs for HCC, are still unknown.

In this study, we found that the expression of CDK5 was significantly increased in HCC and that enhanced CDK5 expression was directly correlated with decreased survival, higher tumor recurrence, and vascular invasion. We then demonstrated that CDK5 promoted HCC progression of HCC in vitro and in vivo by xenograft implantation or diethylnitrosamine (DEN)-induced HCC tumorigenesis models. Mechanistic investigation indicated that CDK5 phosphorylates and stabilizes TPX2 to promote HCC. Since CDK5 activity is vital for hepatocellular carcinoma tumorigenesis, drugs that target CDK5 kinase activity may be a potential curative treatment for HCC. Tamoxifen (TMX), which is an endocrine treatment for breast cancer, has been reported as a new inhibitor of CDK5 kinase activity by interrupting the association between CDK5 and its activator p35/p25 [19]. The administration of TMX significantly inhibited HCC cell proliferation and migration, demonstrating that the kinase activity of CDK5 is vital for HCC progression. It also increases the possibility for personalized medicine in HCC patients with high CDK5 expression. These results determine the critical role of CDK5 in the pathogenesis of HCC and provide novel therapy targets for HCC treatment with CDK5 and its substrates.

## Materials and methods

### Antibodies, regents, and plasmids

Antibodies against CDK5 (C-8), p35 (C-19), and TPX2 were purchased from Santa Cruz Biotechnology (Dallas, TX). Anti-β-actin, anti-GFP, anti-Rb, and pRb-Ser807/Ser811 antibodies were obtained from Cell Signaling Technology (Danvers, MA). Anti-ki67 antibodies and the CDK5 inhibitor roscovitine were acquired from Genetex and Cell Signaling Technology, respectively. TMX was purchased from Sigma. Lentivirus of CMV-EGFP-3FLAG-CDK5, CMV-EGFP-3FLAG-kinase-dead-CDK5, and control were obtained from Neuron Biotech (Shanghai). TPX2 was amplified by PCR and inserted into GFP-C3 to generate GFP-C3-TPX2 vector. GFP-TPX2-S486A and GFP-TPX2-S486D mutations were generated using a site-directed mutagenesis kit following the instruction manual (Stratagene).

### Human tissue samples

Human liver tumor tissues and respective adjacent non-tumor tissues of 90 patients with detailed pathological data and follow-up information were obtained from Zhongshan Hospital of Xiamen University. All samples were collected by hepatectomy from 2010 to 2013. This study was approved by the ethics committee of Zhongshan Hospital of Xiamen University, and written consent was obtained from all patients involved.

### Immunohistochemistry (IHC)

Paraffin-embedded tissue sections (10-μM thick) were immunostained. The tissue sections in paraffin were rehydrated through incubations in xylene and then an alcohol gradient from 100 to 70%. The antigen was retrieved by heating in boiling temperature for 10 min using citrate antigen retrieval solution. The primary antibodies were incubated at 4 °C overnight. CDK5 (Santa Cruz) was used at 1:150. CDK5 was incubated with secondary goat antirabbit IgG (Life Technologies) at room temperature for 1 h and then washed three times with PBS. All slides were analyzed by two individuals trained in IHC, and interpretations were confirmed by a pathologist. Briefly, the CDK5 immunoreactivity score was determined by multiplying the CDK5 staining intensity (scored as 0, no staining;1, weak staining; 2,moderate staining; or 3, strong staining) and the percentage of Cdk5-positive cells (scored as 1, 0–25% positive cells; 2, 26–74% positive cells; 3, 75–89% positive cells; or 4, 90–100% positive cells). The result was considered positive when the product of the two scores was more than 4.

### Cell culture, transfections, and drug treatment

Human HCC cell lines HepG2, Hep3B, sk-Hep1, QGY7701, SMMC7721, Huh7, MHCC97h, and LM3 were purchased from Shanghai Cell Bank (Shanghai, China). All cells were cultured at 37 °C in a humidified atmosphere of 5% CO_2_ in DMEM supplemented with 10% heat-inactivated FBS and Pen/Strep.

To generate CDK5 knockdown stable cell lines, a target set of shRNA sequences directed against human CDK5 was used. Cells were transfected with DNA constructs by TurboFect (Invitrogen) according to the manufacturer’s protocol and were selected with the corresponding antibiotic puromycin at 1 μg/mL (Sigma) 48 h after transfection.

For the construction of CDK5 overexpression stable clones, EGFP-WT-CDK5 and a dominant-negative CDK5 containing a D144N mutation were created using PCR. Two hundred ninety three T-packaging cells were used for lentivirus production. The supernatant containing lentivirus was harvested to transducer and Huh7 cell lines. Resistant clones were selected in the presence of puromycin at 1 μg/mL (Sigma).

### Western blotting and coimmunoprecipitation

Harvested cells were homogenized in ice-cold NP-40 lysis buffer with protease inhibitor mix. The samples were centrifuged at 12,000×g for 12 min at 4 °C. The supernatant was collected and total protein levels were measured using the micro BCA protein assay kit (Thermo Fisher Scientific). For western blotting, the lysates were separated with SDS-PAGE and electrophoretically transferred onto nitrocellulose membranes. The membranes were blocked with 5% nonfat milk in TBST and probed with primary antibodies overnight, followed by treatment with secondary antibodies and ECL western blotting detection reagents. For immunoprecipitation, the cell lysates were incubated with corresponding antibody at 4 °C for 4 h, followed by overnight incubation with protein G. The beads were washed five times with ice-cold NP-40 lysis buffer, and the bound proteins were analyzed using SDS-PAGE and immunoblotting analysis.

### Cell proliferation, counting, migration, and invasion assays

Cells were seeded into 6-cm tissue culture dishes (0.5 × 10^3^ cells per well) and cultured for 14 days. Subsequently, the cells were fixed with absolute ethyl alcohol for 15 min and stained with 1.0% crystal violet for 10 min. The number of colonies formed was counted in 10 different fields. 1.0 × 10^5^ cells were seeded into 24-well plate. The cells were divided into 7 groups, each group 3 wells, cultured for one week. Then collected and counted the number of cells of each group daily. The number of cells in 7 days was plotted as cell growth curve to determine the absolute cell growth rate. The stable transfected SMMC-7721 cells (shRNA and control) were starved for 24 h in serum-free media, and 2 × 10^5^ cells were plated into the upper chamber with 8-mm pores (Corning, Life Sciences, Lowell, MA). DMEM containing 10% FBS was placed in the lower chamber. For migration assay, cells were stained with crystal violet (Sigma) after incubation for 24 h. For invasion assay, the upper chambers were coated with Matrigel (BD Biosciences) and stained after 48 h. A total of 5 fields were counted for each filter. The experiments were performed three times independently.

### Protein half-life assay

SMMC-7721 and Huh7 cells were transfected with control shRNA, CDK5 shRNA, GFP control vector, or GFP-CDK5 vector. After transfection, 100 μg/mL cycloheximide (CHX) was added into the media for the indicated times. The cells were harvested and subjected to immunoblot analysis with antibodies against TPX2, Cdk5, and GAPDH. HEK293T cells transfected with GFP-TPX2-WT, GFP-TPX2-S496A, and GFP-TPX2-S486D were treated with CHX (100 μg/mL) and subjected to immunoblot analysis with antibodies against GFP and GAPDH.

### Tumor xenograft study and induced HCC mouse model

Cdk5 stable knockdown SMMC7721 cells and control cells (3 × 10^6^) were subcutaneously injected into the right flanks of 6-week-old male nude mice. Huh7 cells infected with Cdk5 lentivirus and selected with puromycin were injected into nude mice as the above method. The diameters of tumors were measured every 4 days using calipers. Mice were sacrificed 60 days after injection or when tumor reached a static size. The tumors were weighed and statistically analyzed, and the livers were collected.

A colony of Cdk5+/− mice was maintained on a mixed C57BL/6 J background. To induce HCC, 15-day-old C57/B6 male mice and Cdk5+/− mice were injected i.p. with 25 mg/kg DEN (Sigma). Mice were sacrificed after 9 to 10 months, and tumors larger than 1 mm in diameter on the liver surface were counted. Tumors larger than 5 mm across were dissected for biochemical and molecular analyses. All animal procedures in this study were approved by the Institutional Animal Care and Use Committee of Xiamen University.

### Partial hepatectomy

Cdk5 heterozygous knockout mice (cdk5 +/−) and wild-type mice of 8–10 weeks of age were subjected to a 2/3 partial hepatectomy (PH). The procedure was performed as described previously [20]. Mice were sacrificed at 1, 2, 3 5, 7, and 14 days post PH under anesthesia, and the livers were collected. The ratio of the weight of the remaining liver after PH over the body weight was taken as the liver to body weight ratio. The results obtained were the mean of three different animals per time point.

### In vitro kinase assay

Recombinant GST-TPX2 and GST-TPX2 (S486A) proteins, as well as GST tag protein, were purified from *E. coli* by affinity chromatography. A substrate (1 μg) was added into kinase assay buffer (CST) containing 25 mM Tris-HCl (pH 7.5), 2 mM dithiothreitol (DTT), 5 mM beta-glycerophosphate, 0.1 mM Na_3_VO_4_, and 10 mM MgCl_2_, and incubated with CDK5/p25 kinase and 50 μM ATP-γ-S at 30 °C for 45 min. The samples were alkylated with 2.5 mM PNBM/5% DMSO (Abcam), incubated at room temperature for 1 h, and then subjected to western blotting. Phosphorylated proteins were immunoblotted with an anti-thiophosphate ester antibody.

### Statistical analysis

Clinical parameters were analyzed using the chi-square test. Survival analysis was performed using the Kaplan-Meier method. Student’s t-test or one-way ANOVA was used to determine statistically significant difference between groups. All data were expressed as mean ± SD. Results between groups were considered significant at *P* < 0.05. The different degrees of significance were indicated as follows in the bar graphs: *P < 0.05; **P < 0.01; ***P < 0.001.

## Results

### Enhanced CDK5 levels in HCC tissues are associated with poor prognosis in HCC cancer

**Fig. 1:**
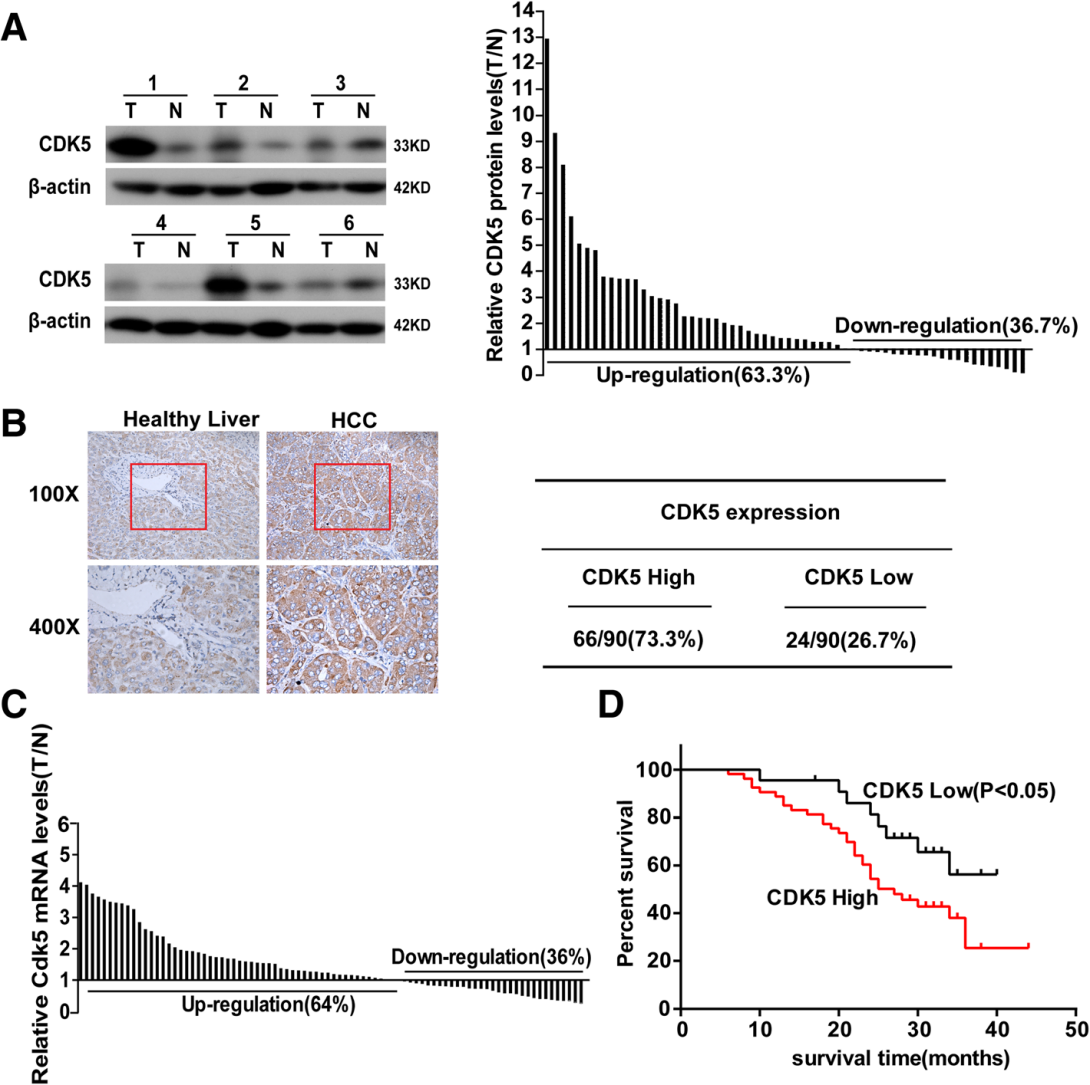
CDK5 is increased in HCC and correlates with postoperative survive. **a** Immunoblotting analysis of CDK5 protein expression in HCC tissue specimens(T) and non-cancerous surrounding tissues(N), The ratios of CDK5 in HCC tumors compared to paired non- cancerous tissue controls (T/N) from 60 patients are presented in the right panel. **b** Immunohistochemistry of CDK5 in HCC tissue specimens and respective non-tumor tissue specimens (NT). Samples were classified into two groups according to CDK5 expression: CDK5 positive group (T > N,66 of the 90(73.3%)) and negative group (T ≤ N,24 of the 90(26.7%)). **c** Real-time PCR analysis of CDK5 mRNA expressionin HCC tumor and corresponding nontumortissues. Ratios of HCC tumor compared to respectivenon-tumor tissue (T/N) from 86 patients are presented. **d** Kaplan-Meier survival curves of “High CDK5” and “Low CDK5” group HCC patients

We examined CDK5 protein levels in HCC tumors and paired adjacent noncancerous tissues from 66 patients by western blotting. We observed that HCC tumor tissues expressed significantly higher levels of CDK5 compared with noncancerous tissues in the majority of patients (63.3%, Fig. 1a). To further evaluate the clinical prognostic role of CDK5 in HCC, we examined an additional 90 samples of HCC patients who underwent surgery at least 5 years ago and with paired detailed pathological scoring record. Immunohistochemistry CDK5 staining was performed, and samples were classified into two groups according to CDK5 expression. Sixty-six of the 90 (73.3%) cases had higher expression levels of CDK5 in tumor tissues than in noncancerous tissues and were classified as “CDK5 high.” The remaining 24 (26.7%) cases with a comparable CDK5 level between tumor and noncancerous tissues were classified as “CDK5 low” (Fig. 1b). The transcription of CDK5 was also examined by real-time qPCR. We found that CDK5 (among 64%) transcripts were higher in tumor tissues than in noncancerous tissues (Fig. 1c). Five-year survival analysis revealed that the “CDK5 high” group had a much shorter survival compared with the “CDK5 low” group (Fig. 1d). In addition, the “CDK5 high” group showed more deleterious clinicopathologic features in vessel invasion and recurrence (Table 1). Tumor recurrence, which is the main factor influencing the effect of surgery, complicates most cases at 5 years after liver resection. Microvascular invasion is a risk factor for immediate post-operative recurrence of HCC [21, 22]. Our results indicated that CDK5 may participate in HCC metastasis, and CDK5 expression level was an independent prognostic factor of HCC.

**Table 1:** Correlations between CDK5 expression and clinical characteristics in HCC patients

	CDK5(T-N)		Total	*P*
	T≦N	T > N		
Age				0.436
< 50	8	28	36	
≥ 50	16	38	54	
Gender				0.511
Male	19	57	76	
Female	5	9	14	
HBV				1.000
Positive	21	56	77	
Negative	3	10	13	
Liver cirrhosis				0.972
Yes	17	47	64	
No	7	19	26	
Tumor size(cm)				0.921
< 5	9	24	33	
≥ 5	15	42	57	
Histilogical differentiation				0.080
Well	4	4	8	
Moderate	16	53	69	
Poor	4	9	13	
Serum AFP				0.873
< 25 μg/l	11	29	40	
≥ 25 μg/l	13	37	50	
Vessel Invasion				0.004^*^
Yes	10	49	59	
No	14	17	31	
Recurrence				0.033^*^
No	17	30	47	
Yes	7	36	43	
Distant Metastasis				0.941
No	18	50	68	
Yes	6	16	22	

^*^indicates *P* < 0.05

### CDK5 promotes HCC cell proliferation, migration, invasion, and motility in kinase activity

**Fig. 2:**
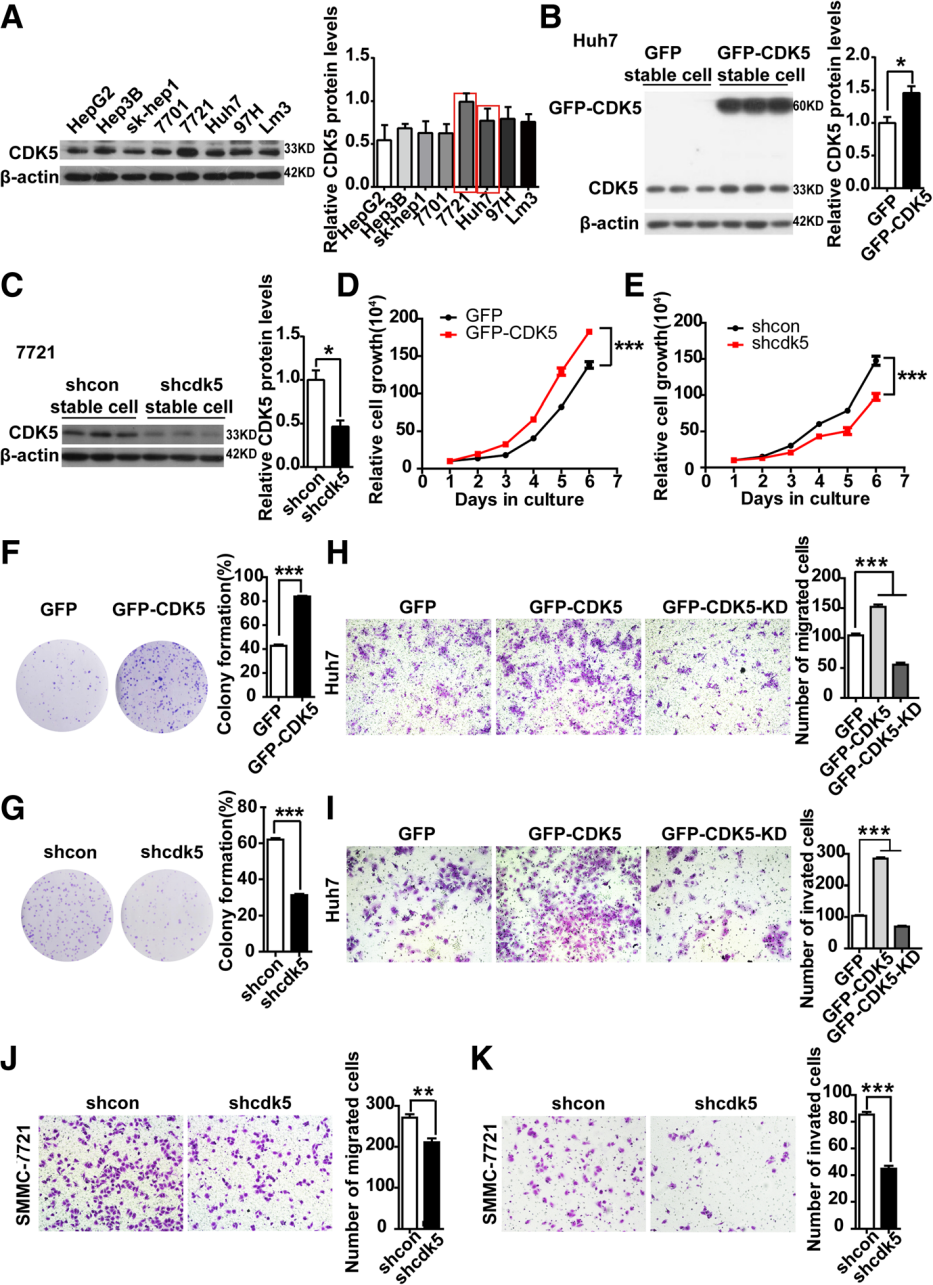
CDK5 related to HCC cell proliferation, migration and invasion. **a** Expression of CDK5 in 8 HCC cell lines by immunoblotting analysis. **b** Immunoblotting analysis of expression of CDK5 protein in Huh7 cells after infection of GFP-N1 and GFP-CDK5 lentivirus. **c** Immunoblotting analysis of expression of CDK5 protein in 7721 cells after transfection of shcdk5 or empty vector. **d** Cell counting assay in CDK5 stably expressing Huh7 cells for 5 days, t-test ***p < 0.001, *n* = 3. **e** Cell counting assay in shCDK5 SMMC-7721 cells for 5 days. t-test ***p < 0.001, *n* = 3. **f** Colony formation assay in CDK5 stably expressing Huh7 cells and control cells, t-test ***p < 0.001, n = 3. **g** Colony formation assay in SMMC-7721 cells after infection of shCDK5 or empty vector, t-test ***p < 0.001, *n* = 3. **h** migration(24 h) and **i** invasion(48 h) assay in Huh7 cells transfected with GFP-CMV, GFP-CDK5,GFP-CDK5-KD constructs; One Way ANOVA on Ranks,*P < 0.05; **P < 0.01;***P < 0.001. **j** migration (24 h) and **k** invasion (48 h) assays in SMMC-7721 cells after infection of shCDK5 or empty vector. The number of migrated and invaded cells were counted and the quantifications were present on right panel. t test,*P < 0.05; **P < 0.01;***P < 0.001

Based on our observation that higher CDK5 expression was found in HCC tumor tissues, we postulated that CDK5 promotes HCC cell growth. To prove this hypothesis, we first confirmed the expression of CDK5 in eight HCC cell lines (Fig. 2a). Huh7 cells with relatively low CDK5 expression were used to generate stable CDK5 overexpression cell line by infecting cells with GFP-tagged CDK5 lentivirus or control GFP virus (Fig. 2b). Meanwhile, SMMC-7721 cells with relatively high CDK5 expression were used to generate stable CDK5 knockdown cell line by infecting cells with lenti-shcdk5 virus and control lenti-sh-control (Fig. 2c). As shown in Fig. 2d, HCC Huh7 cell proliferation was significantly promoted by CDK5 overexpression, whereas knockdown of Cdk5 in SMMC-7721 cells inhibited cell growth (Fig. 2e). Similarly, CDK5 overexpression dramatically enhanced colony formation of Huh7 cells, and CDK5 depletion significantly reduced colony formation of 7721 cells (Fig. 2f and g). Meanwhile, in vivo experiment,  knockdown of CDK5 inhibited liver regeneration after partial hepatectomy  in mice  (Additional file 1: Figure S1). CDK5 was reported to participate in certain cancer cell migration and invasion (8, 16–17). To investigate whether CDK5 was also involved in HCC cell migration and invasion, we overexpressed GFP-tagged CDK5 and GFP-tagged CDK5-KD (kinase death) in Huh7 cancer cell lines. We observed that cell migration, invasion, and wound healing were significantly increased by CDK5 but not CDK5-KD overexpression (Fig. 2h, i; Additional file 2: Figure S2). Conversely, CDK5 depletion dramatically reduced 7721 cell migration, invasion, and wound healing (Fig. 2l, k; Additional file 2: Figure S2). The above data suggest that the function of CDK5 in HCC cell migration, invasion, and motility relies on its kinase activity. To confirm this further, roscovitine, a CDK5 kinase activity inhibitor, was used. We found that roscovitine inhibited HCC cell colony formation and migration in a dose-dependent manner (Additional file 3: Figure S3). These results provide evidence that Cdk5 promotes HCC cell growth and migration relying on its kinase activity.

### CDK5 promotes HCC xenograft tumorigenesis and metastasis in immunocompromised mice

**Fig. 3:**
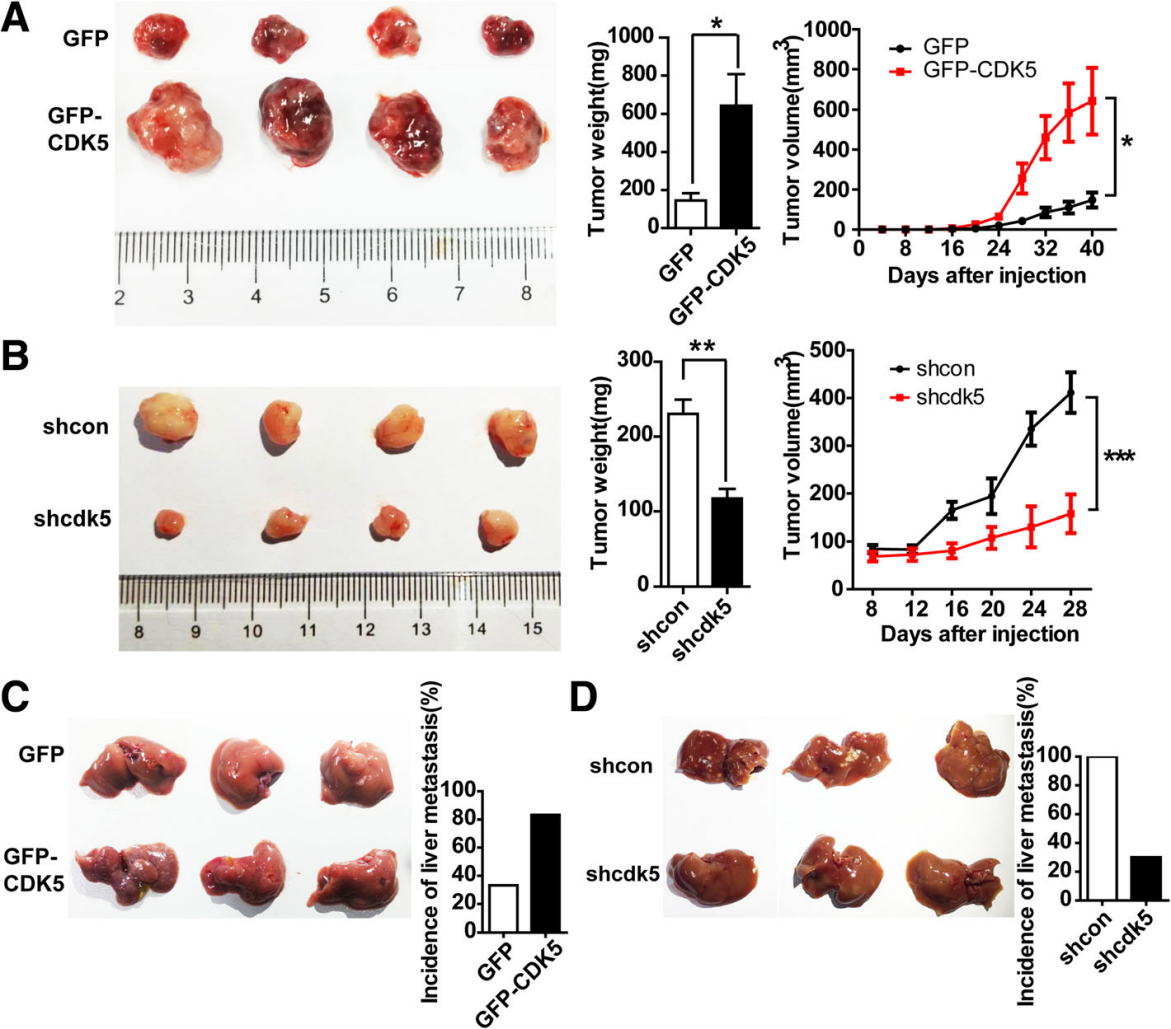
CDK5 promotes HCC cell tumorigenic growth and liver metastasis in xenograft implants. **a** Tumors of GFP-MV and GFP-CDK5 cells grown in nude mouse model; Tumor weight of each group; Tumor volume over the time is shown; t test,*P < 0.05; **P < 0.01;***P < 0.001. **b** Tumors of nt shScb SMMC-7721 and Cdk5 shRNA SMMC-7721 cells grown in nude mouse model; Tumor weight of each group; Tumor volume over the time is shown; t test,*P < 0.05; **P < 0.01;***P < 0.001. **c** Liver metastases of GFP-CMV and GFP-CDK5 Huh7 cells grown in nude mouse was shown; Incidence of liver metastasis in each group of nude mice was shown in the right panel. **d** Liver metastases of shcon and shcdk5 SMMC-7721 cells grown in nude mouse was shown; Incidence of liver metastasis in each group of nude mice was shown in the right panel

Based on our HCC cell proliferation and migration data in vitro, we hypothesized that CDK5 promotes HCC tumorigenesis and metastasis in vivo. To further verify this hypothesis, we performed xenograft experiments by subcutaneously injecting the stable CDK5 overexpression HCC Huh7 cells into immunocompromised mice. Tumors established from CDK5 overexpression Huh7 cells were significantly larger than the control tumors (Fig. 3a). More interestingly, in low invasive Huh7 cell lines, more than 80% of nude mice injected with GFP-CDK5-Huh7 cells developed liver metastasis compared with GFP-Huh7 control cells (Fig. 3c). By contrast, in high invasive 7721 cell lines, xenografting using sh-Cdk5–7721 cells resulted in smaller tumor formation (Fig. 3b) and less liver metastasis (Fig. 3d) compared with sh-control-7721 cells in immunocompromised mice. These results strongly support the notion that CDK5 can promote HCC tumorigenesis.

### Half depletion of CDK5 reduces HCC tumor development in DEN-induced HCC mice

**Fig. 4:**
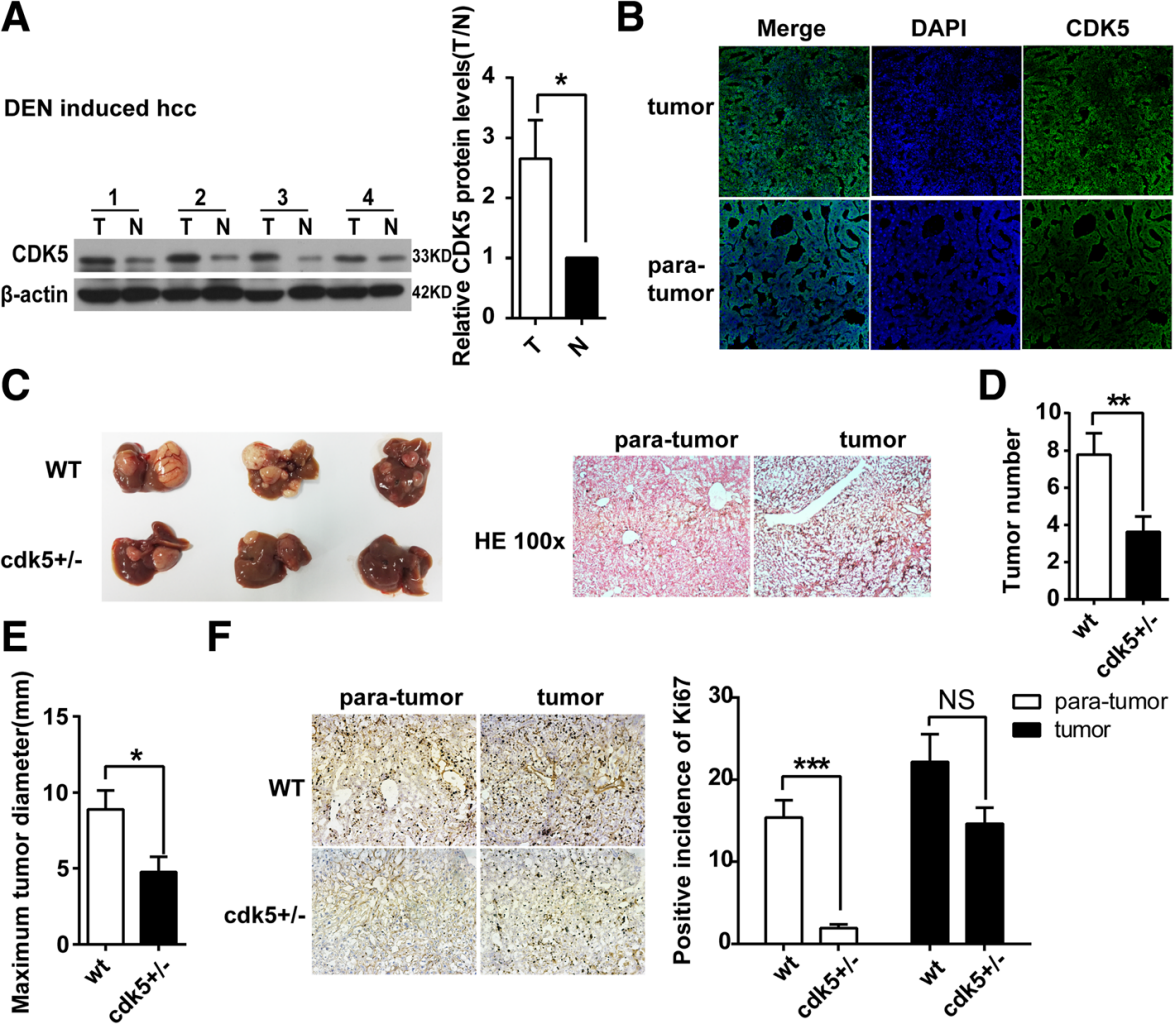
Half depletion of CDK5 reduces HCC tumor development in DEN-induced HCC mice. **a** Immunoblotting analysis of CDK5 protein in tumor(T) and non-cancerous surrounding tissues(N) of DEN induced HCC mouse model. t test,*P < 0.05. **b** Immunofluorescence of CDK5 in DEN induced HCC mouse model. **c** Specimens of liver obtained from DEN induced HCC model in wild type and half depletion of CDK5 mice after 10 months injection. Hematoxylin-eosin staining of tumor and corresponding para-tumor liver tissue was shown in the right panel. **d** Tumor numbers after 10 months injection of DEN was shown. t test, **P < 0.01. **e** Maximum diameters of tumor was shown, t test,*P < 0.05. **f** Immunostainings of tumor and para-tumor liver tissue staining for Ki67 in wild type and half depletion of CDK5 mice. Positive incidence of Ki67 was shown in the right panel. t test, ***P < 0.001

DEN, a hepatic procarcinogen, is widely used to induce HCC in mice. Consistent with our observations in human HCC tumor tissues, the expression levels of CDK5 were also highly upregulated in DEN-treated livers compared with control livers by western blotting and immunostaining (Fig. 4a and b). To further investigate the effects of CDK5 on DEN-induced HCC and in view of the embryonic lethality of CDK5 homozygous knockout mice, WT mice and Cdk5 heterozygous knockout mice (Cdk5^+/−^) were treated with DEN injection. HE staining indicated HCC tumorigenesis in DEN-induced mice (Fig. 4c). We found that the tumor size and numbers in DEN-induced *Cdk5*^*+/−*^ mice were much lesser than those in WT mice (Fig. 4c, d, e). Tumor cell growth was also significantly decreased as observed using Ki67 staining in DEN-induced Cdk5^+/−^ mice compared with WT mice (Fig. 4f).

### TMX suppresses HCC cell growth and xenograft tumorigenesis by inhibiting CDK5 kinase activity

**Fig. 5:**
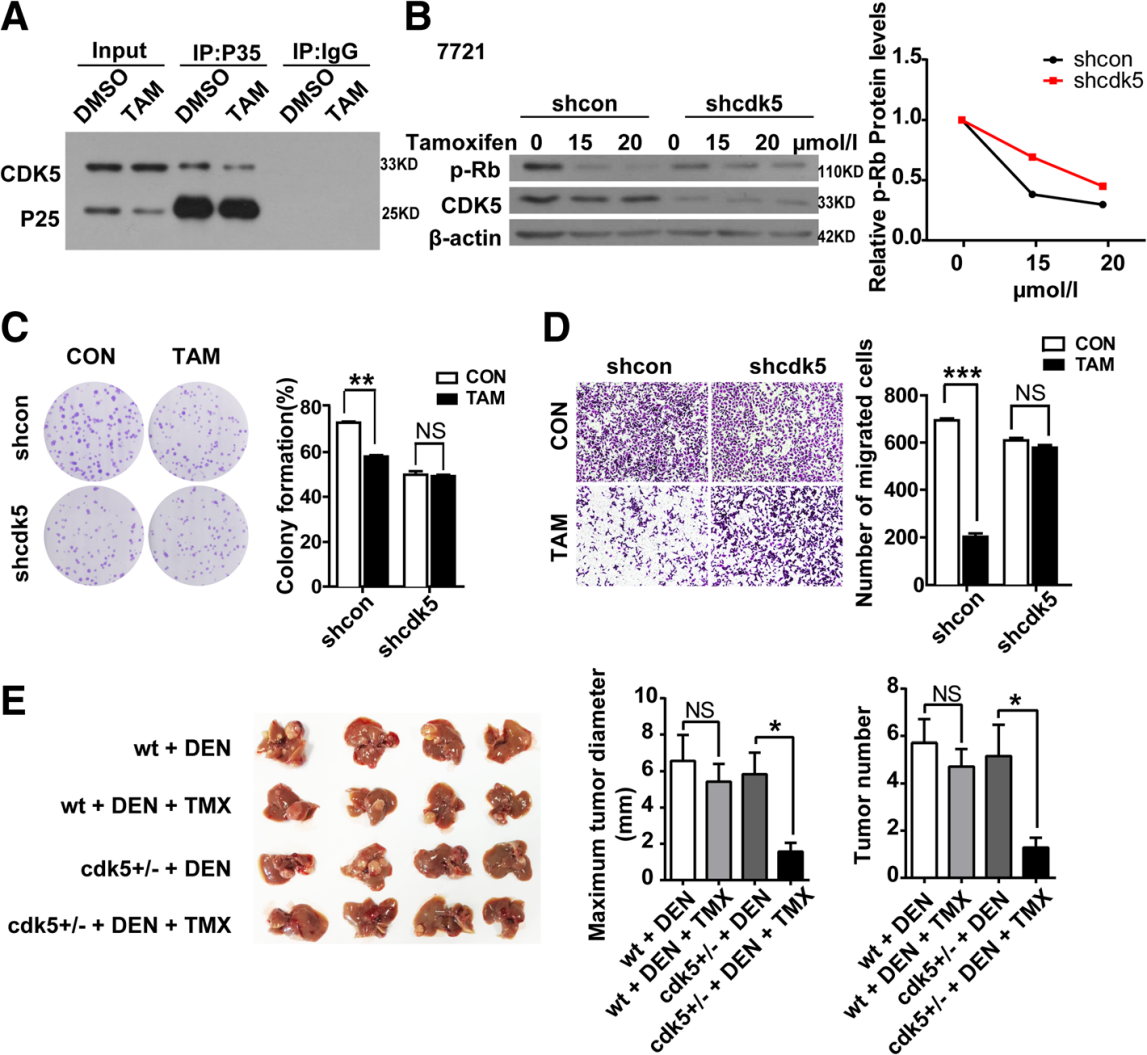
Tamoxifen induced apoptosis and inhibited HCC cell growth and migration by intervening in CDK5/p25Interaction. **a** cells transfected with GFP-CDK5 and GFP-P25, co-treated with DMSO or TMX (20 μM). The extracts were then immunopurified using anti-P35 antibody and analyzed by western blotting using antibodies directed against GFP. *P < 0.05; **P < 0.01;***P < 0.001. **b** Immunoblotting analysis of phospho-Rb(P-Rb) and Cdk5 expression in shcon SMMC-7721 and cdk5 shRNA SMMC-7721 cells treated with increasing concentrations of TMX for 24 h, relative p-Rb protein level over the time is shown. **c** Cell Proliferation Assay of shcon SMMC-7721 and shcdk5 SMMC-7721 cells treated with TMX(10 μmol/l) for 24 h. **d** Migration (24 h) assays in shcon SMMC-7721 and cdk5 shRNA SMMC-7721 cells treated with TMX (10 μmol/l) for 24 h. **e** HCC mice induced by DEN were injected i.p.with TMX (Sigma) dissolved in corn oil at 100 mg/kg per daily dose. 2 months after the final injection of three, animals were euthanized and tumors bigger than 1 mm in diameter on the liver surface were counted. Maximun diameter and number of tumor were shown in the right panel. t test,*P < 0.05

As our observation above, CDK5 promoted HCC tumorigenesis dependent on kinase activity in vitro and in vivo. Thus, drugs aim to inhibit CDK5 kinase activity may be potential for HCC treatment. TMX, which is the most prescribed hormone therapy for breast cancer, is a new CDK5/p25 interaction blocker and suppresses CDK5 activity eventually [19, 23, 24]. We first confirmed that TMX can disassociate the CDK5-P25 interaction and inhibit CDK5 kinase activity measured by Rb(retinoblastoma) phosphorylation, which is a marker for CDK5 kinase in HCC cells (Fig. 5a, b). To explore the effect of TMX in HCC development and determine whether CDK5 is involved in the progression, TMX was administered to stable CDK5-deficient and control 7721 cells. About 10 μm/L TMX significantly decreased colony formation and migration in control 7721 cells, but this effect was abolished in CDK5-deficient 7721 cells (Fig. 5c, d). These data suggest that TMX could also inhibit HCC cell growth and migration in vitro. To further study the effect of TMX on DEN-induced HCC in vivo, mice were treated with corn oil or corn oil-dissolved TMX daily for two weeks following DEN injection. This treatment significantly decreased the tumor growth and number of HCC in DEN-challenged *cdk5*^*+/−*^ mice (Fig. 5e).

### CDK5-dependent phosphorylation mediates stabilization of TPX2

**Fig. 6:**
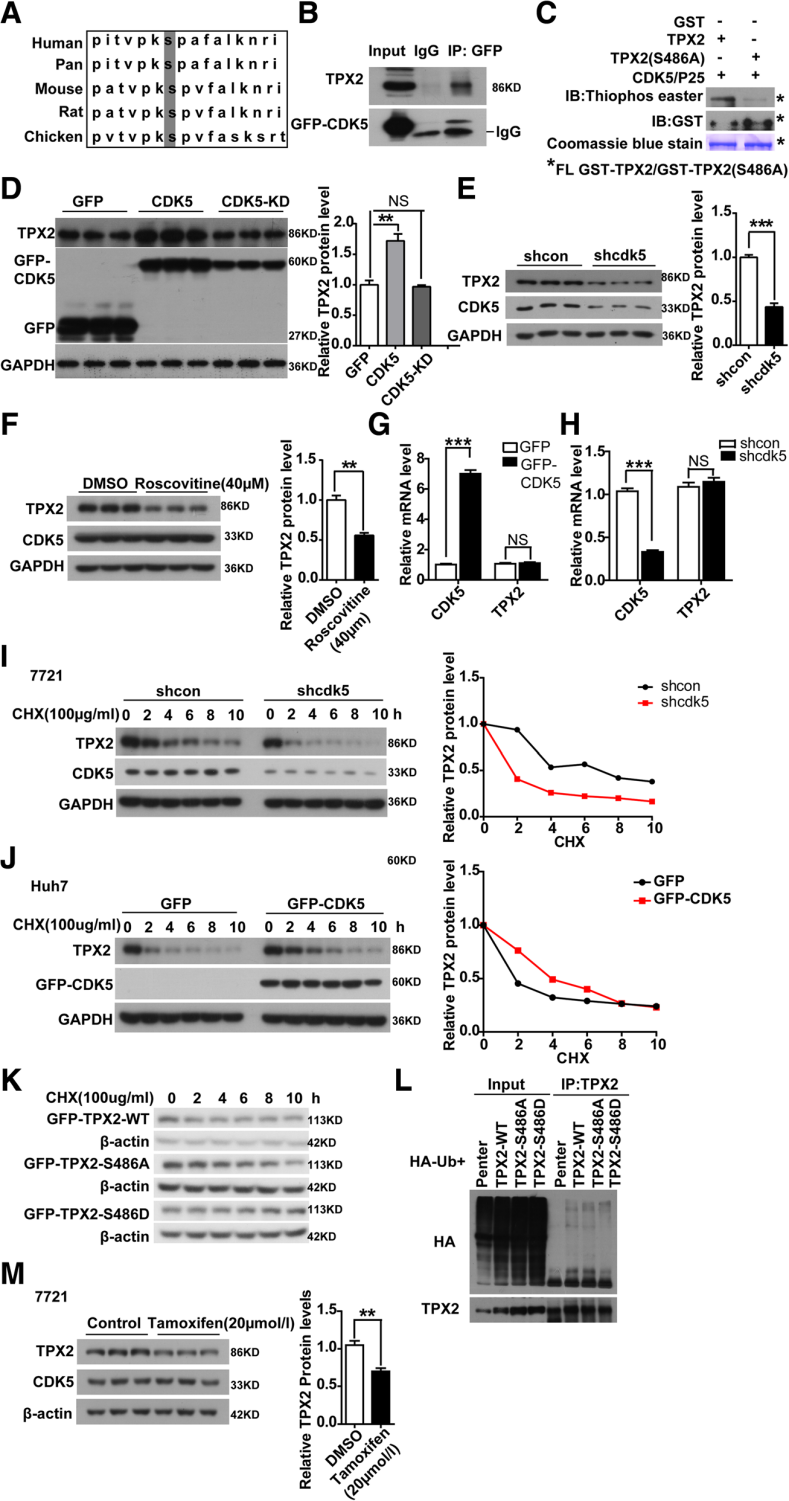
CDK5 modulates protein stability of TPX2. **a** The putative consensus CDK5 phosphorylation sites of TPX2 proteins in different organisms. Alignment of TPX2 proteins sequence from different organisms indicated that the CDK5 phosphorylation site is conserved in different organisms. **b** Protein complex between TPX2 and CDK5. Cell lysates were immunoprecipitated (Co-IP) with GFP antibody followed by IB with TPX2 antibodies. **c** In vitro phosphorylation assay of recombinant wild-type TPX2 and non-phosphorylatable TPX2 (S486A) with activated CDK5/P25. Immunoblot detecting the phosphorylation of purified GST recombinant TPX2, and non-phosphorylatable TPX2-S486A mutation using an antibody against thiophosphate esters (Top). Coomassie blue stain (bottom) and the immunoblot with anti-GST antibodies (middle) reveals that both wild-type TPX2 and non-phosphorylatable TPX2(S486A) were loaded in similar amounts. The asterisk indicated that the f​ull length of TPX2-WT and TPX2-S486A. **d** Immunoblotting of TPX2 are shown in Huh7 cells transfected with GFP-CDK5-CMV, GFP-CDK5, GFP-CDK5-KD constructs, GAPDH serve as control. The average of TPX2 protein levels with Mean values±SEM are presented in the right panel, One Way ANOVA on Ranks,*P < 0.05; **P < 0.01;***P < 0.001. **e** Similar to **d**, TPX2 protein levels in shCDK5 SMMC-7721 cells were shown, t test,***P < 0.001. **f** Treatment of SMMC-7721 cells with 40 μM roscovitine inhibited TPX2; The average of TPX2 protein levels with Mean values±SEM are presented in the right panel,t test,**P < 0.01. **g** Relative mRNA levels of TPX2 were measured in CDK5 overexpressed Huh7 cells by Real-Time qPCR, t test,***P < 0.001. **h** Relative mRNA levels of TPX2 were measured in shCDK5 SMMC-7721 cells by Real-Time qPCR, t test,***P < 0.001. **i** Degradation of TPX2 protein in shcon and shCDK5 SMMC-7721 cells after treatment by CHX in in time gradient, relative TPX2 protein level over the time was shown in the right panel. **j** Degradation of TPX2 protein in CDK5 overexpression Huh7 cells after treatment by CHX in in time gradient, relative TPX2 protein level over the time was shown in the right panel. **k** Degradation of TPX2 protein in GFP-CDK5-WT(wildtype), mutant GFP-TPX2 (S486A), phosphor mimic mutant GFP-TPX2 (S486D) after treatment by CHX in in time gradient. **l** In vitro ubiquitination assay was performed in 293 T cells transfected with TPX2-WT,TPX2-S486A, TPX2-S486D, Immunoblotting with anti-HA antibody(above) and anti-TPX2 antibody (below) were shown. **m** Treatment of SMMC-7721 cells with 20 μM tamoxifen inhibited TPX2; The average of TPX2 protein levels with Mean values±SEM are presented in the right panel, t test,***P < 0.001

To identify the mechanism underlying the effect of active CDK5 promoting HCC development, we performed stable isotope labeling by amino acids in cell culture (SILAC) to screen substrates of CDK5 in tumor cells. The oncoprotein TPX2 [25–27], a key regulator for certain tumor progression, attracted our attention. The SILAC assay data indicated that CDK5 could phosphorylate TPX2 at serine 486, which is also conserved among species (Fig. 6a). To further verify this, we first investigated the physical interactions between CDK5 and TPX2. By coimmunoprecipitation assay, TPX2 was found to be associated with CDK5 (Fig. 6b). Subsequently, we performed an in-depth examination to determine whether TPX2 served as a CDK5 substrate and phosphorylation site using in vitro kinase assay. Wild-type and mutant GST-TPX2 (S486A) proteins were purified and incubated with activated CDK5/p25 and ATP-γ-S. If the GST-tagged proteins could be phosphorylated by CDK5/p25, the substrates could be alkylated by PNBM and detected with a thiophosphate ester antibody [28]. As shown in Fig. 6c, thiophosphate ester antibody could detect the phosphorylation band in the presence of CDK5/p25 with WT-TPX2 but not with the mutant TPX2 (S486A). We then investigated the functional effect of CDK5 on TPX2 phosphorylation. Interestingly, WT-CDK5 but not kinase-dead CDK5 can upregulate TPX2 protein level in HCC cells (Fig. 6d). Genetic or pharmaceutical blockade of CDK5 can significantly reduce TPX2 protein level (Fig. 6e, f, Additional file 3: Figure S3, Additional file 5: Figure S5), but it has no effect on its mRNA level (Fig. 6g, h). To further determine the effect of CDK5 on the protein stability of TPX2, we performed CHX treatment to measure the protein stability of TPX2. CDK5 overexpression significantly increased TPX2 protein stability, and the degradation of TPX2 was much faster in Cdk5-deficient condition (Fig. 6i, j). Given that replacement of this serine residue with alanine efficiently blocks CDK5-mediated TPX2 phosphorylation, we investigated whether the phosphorylation event was related to the CDK5-mediated stabilization of TPX2. We then generated phosphor-mimic mutant TPX2 (S486D). The degradation rate of TPX2 (S486D) was much lesser than that of wild-type TPX2 or TPX2 (S486A) (Fig. 6k). The greater protein stability of TPX2 (S486D) may be attributed to less ubiquitination compared with WT-TPX2 (Fig. 6l). These data suggest that phosphorylation of residue S486 plays an important role in CDK5-mediated posttranslational regulation of TPX2. Meanwhile, in our previous study, TMX could inhibit Cdk5 and P25 interaction and decrease the protein level of TPX2 (Fig. 6m, Additional file 4: Figure S4).

### CDK5 promotes HCC development through TPX2

**Fig. 7:**
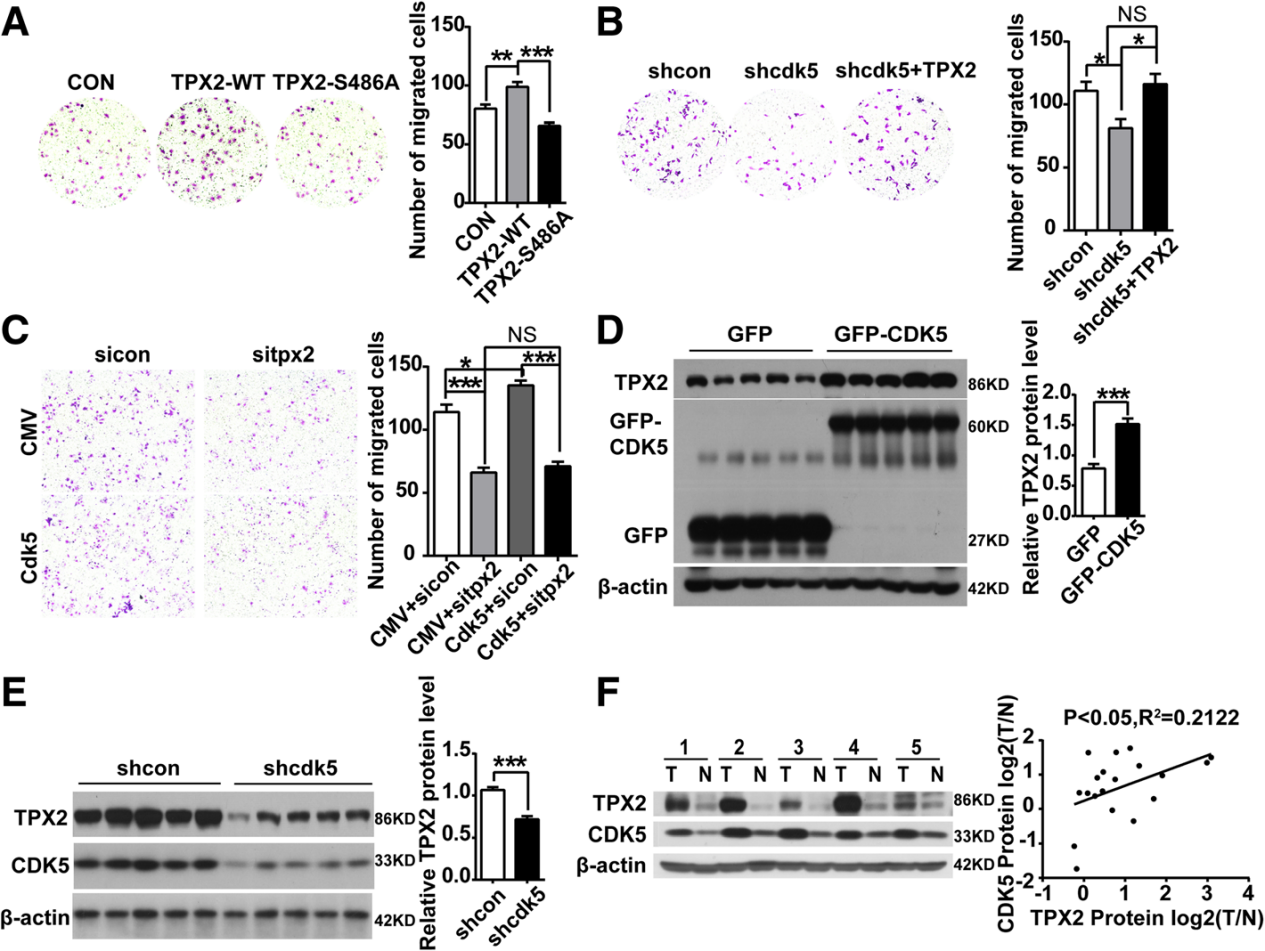
CDK5 promotes HCC development through TPX2. **a** Migration(24 h) assay in SMMC-7721 cells transfected with GFP, GFP-wild-type TPX2 and non- phosphorylatable TPX2 (S486A) constructs; One Way ANOVA on Ranks, **P < 0.01; ***P < 0.001. **b** Migration(24 h) assay in SMMC-7721 cells transfected with shcon, shcdk5 and shcdk5 + TPX2. One Way ANOVA on Ranks, *P < 0.05. **c** Migration(24 h) assay in Huh7 cells transfected with CMV + sicon, CMV + sitpx2, Cdk5 + sicon, Cdk5+ sitpx2, One Way ANOVA on Ranks,*P < 0.05; **P < 0.01;***P < 0.001. **d** Immunoblotting analysis of TPX2 protein in xenograft tumors of CDK5 overexpression stable HCC Huh7 cells and control cells. **e** Immunoblotting analysis of TPX2 protein in xenograft tumors of shcon and shcdk5 stable cells. **f** Immunoblotting analysis of TPX2 and CDK5 protein in Human HCC tissues specimens(T) and non-cancerous surrounding tissues(N). Correlation analysis of TPX2 and CDK5 protein in Human HCC tissues was presented in the right panel

Our previous study showed that CDK5 is related to HCC cell proliferation, migration and invasion, but its mechanism and downstream targets remain unclear. TPX2 has been reported to correlate with tumor migration and invasion [26, 29]. As mentioned above, CDK5 regulates TPX2 and increases its stability, phosphorylation of residue S486 plays an important role in CDK5-mediated posttranslational regulation of TPX2.Transwell assay was performed in TPX2 overexpression and mutant of TPX2. TPX2-WT showed promoting effect on SMMC-7721 cell migration, whereas TPX2-S486A had no effect on migration (Fig. 7a). We also found that decreased migration induced by shcdk5 in SMMC-7721 was rescued with TPX2 overexpression (Fig. 7b). Meanwhile, cell counting and migration assays were also performed in CDK5 overexpressed Huh7 cells with specific siRNA targeting TPX2. We found that decreased proliferation and migration by siRNA targeting TPX2 was not affected with CDK5 overexpression. Increased proliferation and migration by CDK5 was rescued in TPX2 knockdown cells (Fig. 7c, Additional file 6: Figure S6).

TPX2 was also upregulated in tumors derived from xenograft by CDK5 overexpression stable HCC Huh7 cells (Fig. 7d, e). Moreover, in human HCC tissues, CDK5 protein levels were significantly correlated with TPX2 protein levels by western blotting (Fig. 7f). Similarly, in the partial hepatectomy experiment, the protein  levels of TPX2 were subsequently increased after CDK5 (Additional file 1: Figure S1).

## Discussion

Effective treatment of HCC has changed greatly with the development of surgical techniques and new targeted drugs within the past few years. Improved outcomes are still necessary because of its difficulty to diagnose in early stages and high recurrence rates after resection. Thus, improving our understanding of its molecular pathogenesis and identification of novel targets for advanced stage HCC is important.

CDK5 is a serine/threonine kinase that regulates important processes in the nervous system. Since it was discovered more than 20 years ago [30], its function and regulation have been explored in several biological processes and diseases. In recent years, an increasing number of studies have focused on CDK5 and its substrates in cell cycle regulation and cancer. CDK5 has been reported to participate in growth and metastasis of several tumors, such as breast cancer, gastric cancer, lung cancer, liver cancer, pancreatic cancer, prostate cancer, medullary thyroid carcinoma, and myeloma.

CDK5 is an atypical member of CDKs, and its function is not involved in cell cycle control [6, 31]. Several studies have shown that CDK5is active not only in neurons, but also in non-neuronal tissues, including cancer cells [32]. Pozo et al. [15] found that CDK5 is highly expressed in human medullary thyroid carcinoma (MTC) and CDK5 signaling via Rb as target to MTC tumorigenesis and progression. Rea et al. reported that Cdk5 induces phosphorylation of FAK at Ser732, contributing to spindle formation and mitosis of tumor cells [33]. However, the potential role of active CDK5 and its precise molecular mechanisms in HCC remains largely unclear. Our study are strongly congruent with Ehrlich et al. [16] reported that CDK5 promotes progression of HCC, but their study have not involved the active CDK5 vital role and its substrates effect on HCC. In this study, we confirmed that CDK5 is increased in HCC tissues and is related to tumor recurrence, vessel invasion, and survival of patients. Furthermore, we showed that CDK5 promotes cell growth in HCC cell lines. The correlation between CDK5 and migration was reported by Liebl et al., who confirmed that CDK5 controls actin cytoskeleton that supports migration in endothelial cells [34]. The role of CDK5 in HCC cell migration and invasion has not been previously reported. We showed for the first time that active CDK5 promotes cell migration and invasion in HCC cells. Additionally, CDK5 promotes liver metastasis in subcutaneous xenotransplanted tumor model, demonstrating its effect in vivo. We further investigated the Cdk5 knockout (*Cdk5+/−)* transgenic mice with DEN-induced tumor model. A decreased tumor number and size were found in Cdk5-deficient mice, which proved our hypothesis in vivo. Furthermore, to eliminate other pathways of CDK5 in cell proliferation, such as cell cycle and DNA damage, chemotherapy and radiation treatment of HCC cells were performed. We found that there was no change of CDK5 expression in HCC cells after radiation treatment. Meanwhile, the inhibition effect of cell proliferation by chemotherapy and radiation treatment was not related to CDK5 expression (Additional file 4: Figure S4). These findings indicated that the effect of CDK5 in HCC cells may rely on its kinase activity.

Subsequently, we demonstrated that kinase activity of CDK5 is necessary for HCC both in vitro and in vivo (Fig. 5). Thus, the targets and pharmacological inhibition of CDK5 will be interesting for further exploration. TMX, a non-steroidal anti-estrogen drug used in breast cancer, has been used in clinical practice of HCC for decades [35, 36]. However, the effect of TMX in prolonging survival of patients with HCC is controversial. A randomized controlled trial in advanced HCC reported that patients without major hepatic insufficiency seem to achieve some survival benefits [24]. TMX has recently been found to inhibit activity of CDK5 by blocking the CDK5/p25 interaction [19]. In this study, we show that TMX inhibits HCC cell growth and migration in a CDK5-dependent manner, implying a combination of active Cdk5 and TMX as a therapeutic option of HCC.

TPX2, which is critical for mitosis and spindle assembly, has been studied as a marker in various tumors [26, 37–39]. TPX2 is overexpressed in numerous types of cancer, and TPX2 expression level correlates with poor prognosis [40]. Aguirre-Portoles et al. found that TPX2 increases susceptibility to spontaneous lymphomas and lung tumors by maintaining genomic stability, and TPX2 deregulation might act as a driving force of tumor development [26]. TPX2 may serve as a prognostic marker and promotes tumorigenesis and metastasis of HCC [41]. Another study reported that TPX2 expression is associated with proliferation, apoptosis, and EMT in HCC [42]. Meanwhile, numerous studies suggest that TPX2 may be a target for cancer treatment [25, 43]. CDK1/2 phosphorylates TPX2 in vitro and in vivo, and phosphorylation of TPX2 regulates its localization and impacts spindle assembly via Aurora A and Eg5 [44]. Our previous SILAC data showed that TPX2 is a new substrate of CDK5, and its phosphorylation site is serine 486. In this study, we raise a question whether CDK5 signaling and TPX2 exist in HCC. Previous study showed that TPX2 is overexpressed in HCC tissues [43]. Similarly, in our study shown that TPX2 is significantly increased in HCC tissues, and a positive correlation of CDK5 and TPX2 in protein level, that implying a potential regulation of TPX2 by CDK5. In addition, we also found that CDK5 regulated the protein level of TPX2 by improving protein stability. Inhibitors of CDK5 kinase activity, including roscovitine and TMX, can dramatically decrease the protein level of TPX2 in HCC cells. Furthermore, inhibitions of protein stability and migration were observed in TPX2-S486A mutant compared with wild-type and S486D. CDK5 deficiency-induced inhibition of migration was significantly restored by forced overexpression of TPX2. Consistent with this finding, the promote effect of CDK5 in HCC migration disappeared in case of TPX2 deficiency, suggesting a TPX2-dependent manner of CDK5 in HCC migration.

Although the correlation of CDK5 and tumor has been reported for years, this study is the first to comprehensively show active CDK5 and its signaling pathway in tumorigenesis, development, and clinical outcomes of HCC. CDK5 was increased in HCC and correlated with tumor growth, migration, and invasion. The kinase activity of CDK5 was necessary for tumor progression, and the promoting effect of CDK5 was dependent on its substrate TPX2. The therapeutic value of TMX was also presented in this study by regulating CDK5. Taken together, these findings suggest that CDK5may be a potential biomarker and target for molecular therapy in patients with HCC.

## Conclusion

Our study comprehensively demonstrates the function and underlying mechanism of active CDK5 in tumorigenesis and development of HCC. We also identifies TPX2 is an important target of CDK5 which is also high expressed in HCC tumors. Our findings pave the way for searching potential pharmacological targets of CDK5 and its substrates in HCC therapy.

## Additional files

Additional file 1: **Figure S1.** Knockdown of cdk5 inhibits liver regeneration after partial hepatectomy. **a** Percentage of residual liver weight to whole body weight in 1, 2, 3, 5, 7 days after partial hepatectomy. **b** Immunohistochemistry of Ki67 in remnant liver tissue specimens of 3 days after partial hepatectomy. **c** Protein levels of CDK5,TPX2 and P-Rb in 1, 2, 3, 5, 7 days after partial hepatectomy were measured by Western blotting in residual liver tissues. Relative protein level of CDK5 and TPX2 was presented in the right panel. (TIF 9947 kb)

Additional file 2: **Figure S2.** CDK5 is associated with HCC cell migration. **a** The ability of cell motility was compared in shcon and shcdk5 cells by wound healing assay; Light microscopicimages were taken at 0, 24, 72 and 96 h. **b** Wound-healing assays were performed in GFP,GFP-Cdk5, GFP-CDK5-KD Huh7 cells; Light microscopicimages were taken at 0, 24, 72 and 96 h. (TIF 8749 kb)

Additional file 3: **Figure S3.** HCC cell proliferation and migration were inhibited by roscovitine. **a** Colony formation assay inSMMC-7721 cells treated with roscovitine concentration gradient of 10 μmol/l,30 μmol/l,50 μmol/l; One Way ANOVA on Ranks:ns-no statistical differences,***p < 0.001, *n* = 3. **b** migration (24 h) assays in SMMC-7721 cells after treatment of roscovitine (30 μmol/l,50 μmol/l);One Way ANOVA on Ranks: ***p < 0.001, *n* = 5. c Protein levels of TPX2 and p-RB in SMMC-7721 cells after treatment of concentration gradient roscovitine; The average of relative TPX2 protein levels are presented in the right panel. (TIF 15418 kb)

Additional file 4: **Figure S4.** CDK5 has no effect on radiation and chemotherapy induced HCC cell death. **a** Inhibiton effect of oxaliplatin (40 mg/l) in shcon and shcdk5 SMMC-7721 cells by CCK8 assay. **b** Inhibiton effect of arsenic trioxide (ATO,6 μmol/l) in shcon and shcdk5 SMMC-7721 cells by CCK8 assay. **c** PARP and Cdk5 protein levels were measured in SMMC-7721 cells after radiation treatment of 6Gy. **d-e** CCK8 assays in shcdk5 and Cdk5 over-expressed cells compared with control cells after radiation treatment of 6Gy. **f** Colony formation assay after radiation treatment in shcon and shcdk5 SMMC-7721 cells. (TIF 11506 kb)

Additional file 5: **Figure S5.** CDK5 inhibitor reduces TPX2 protein level. **a** Treatment of SMMC-7721 and Huh7 cells with tamoxifen by concentration gradient, TPX2 and Cdk5 protein levels were measured by Immunoblotting,TPX2 protein levels arepresented in the right panel.**b** Similar to **a**, SMMC-7721 and Huh7 cells were treated with roscovitine by concentration gradient, TPX2 andp-Rb protein levels were measured by Immunoblotting, TPX2 protein levels arepresented in the right panel. (TIF 12532 kb)

Additional file 6: **Figure S6.** Cell counting assay in Huh7 cells transfected with CMV + sicon, CMV + sitpx2, Cdk5 + sicon, Cdk5+ sitpx2, One Way ANOVA on Ranks,*P < 0.05; **P < 0.01;***P < 0.001. (TIF 32 kb)

## Data Availability

Please contact the corresponding author for all data requests.

## Abbreviations

CDK5: Cyclin-dependent kinase 5
CHX: Cycloheximide
DEN: Diethylnitrosamine
EMT: Epithelial-mesenchymal transition
HCC: Hepatocellular carcinoma
SILAC: Stable isotope labeling by amino acids in cell culture
TMX: Tamoxifen
TPX2: Target protein for Xklp2
WT: Wild-type
