# Supplementary figures and images for: CDK5-mediated phosphorylation and stabilization of TPX2 promotes hepatocellular tumorigenesis
Source: J Exp Clin Cancer Res. 2019 Jul 4;38:286. doi: 10.1186/s13046-019-1297-6 (PMC6610961; doi:10.1186/s13046-019-1297-6)

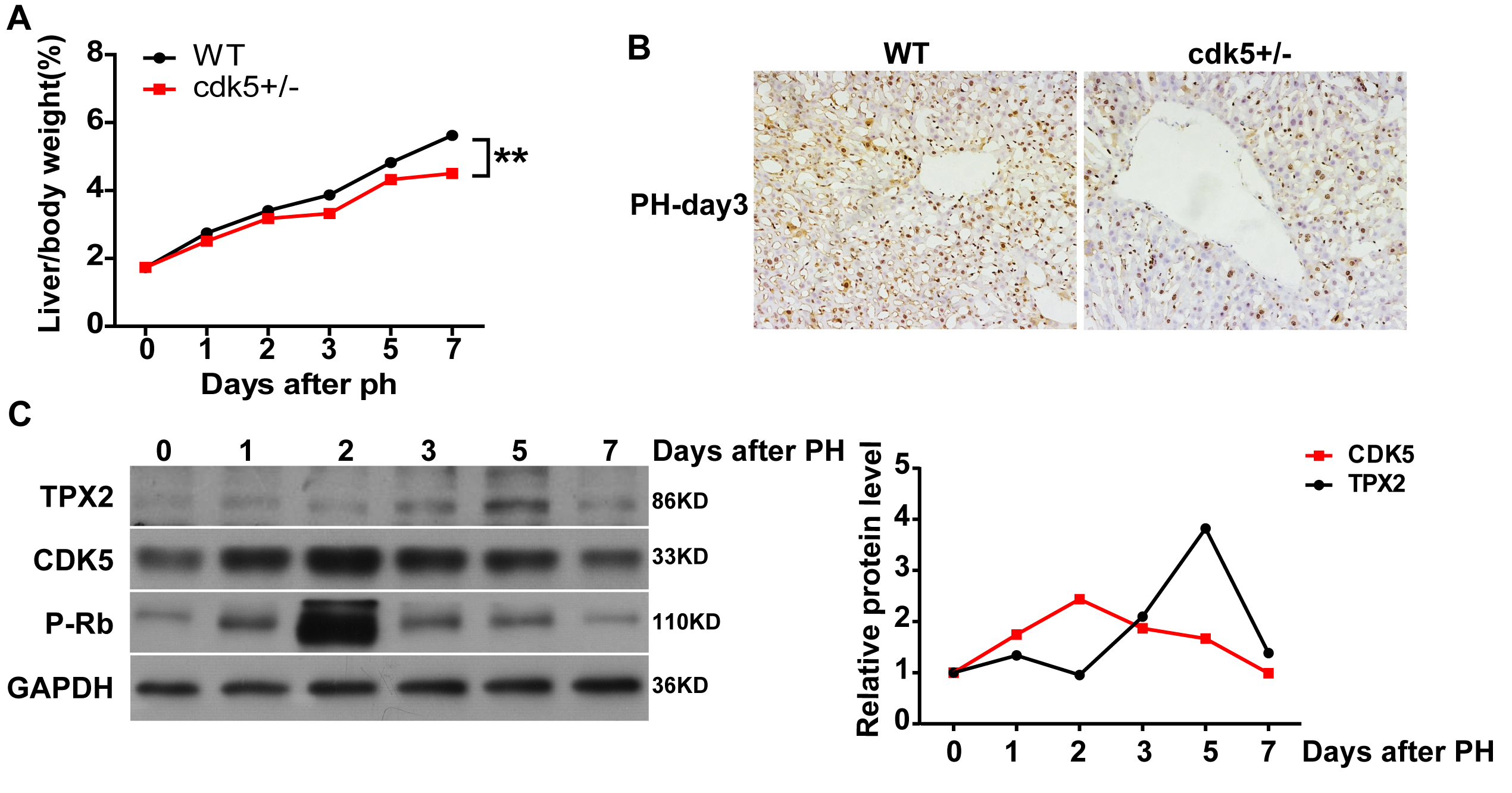

Supplement: Supplementary file 1 — Figure S1. Knockdown of cdk5 inhibits liver regeneration after partial hepatectomy. a Percentage of residual liver weight to whole body weight in 1, 2, 3, 5, 7 days after partial hepatectomy. b Immunohistochemistry of Ki67 in remnant liver tissue specimens of 3 days after partial hepatectomy. c Protein levels of CDK5,TPX2 and P-Rb in 1, 2, 3, 5, 7 days after partial hepatectomy were measured by Western blotting in residual liver tissues. Relative protein level of CDK5 and TPX2 was presented in the right panel. (TIF 9947 kb) [file 13046_2019_1297_MOESM1_ESM.tif]

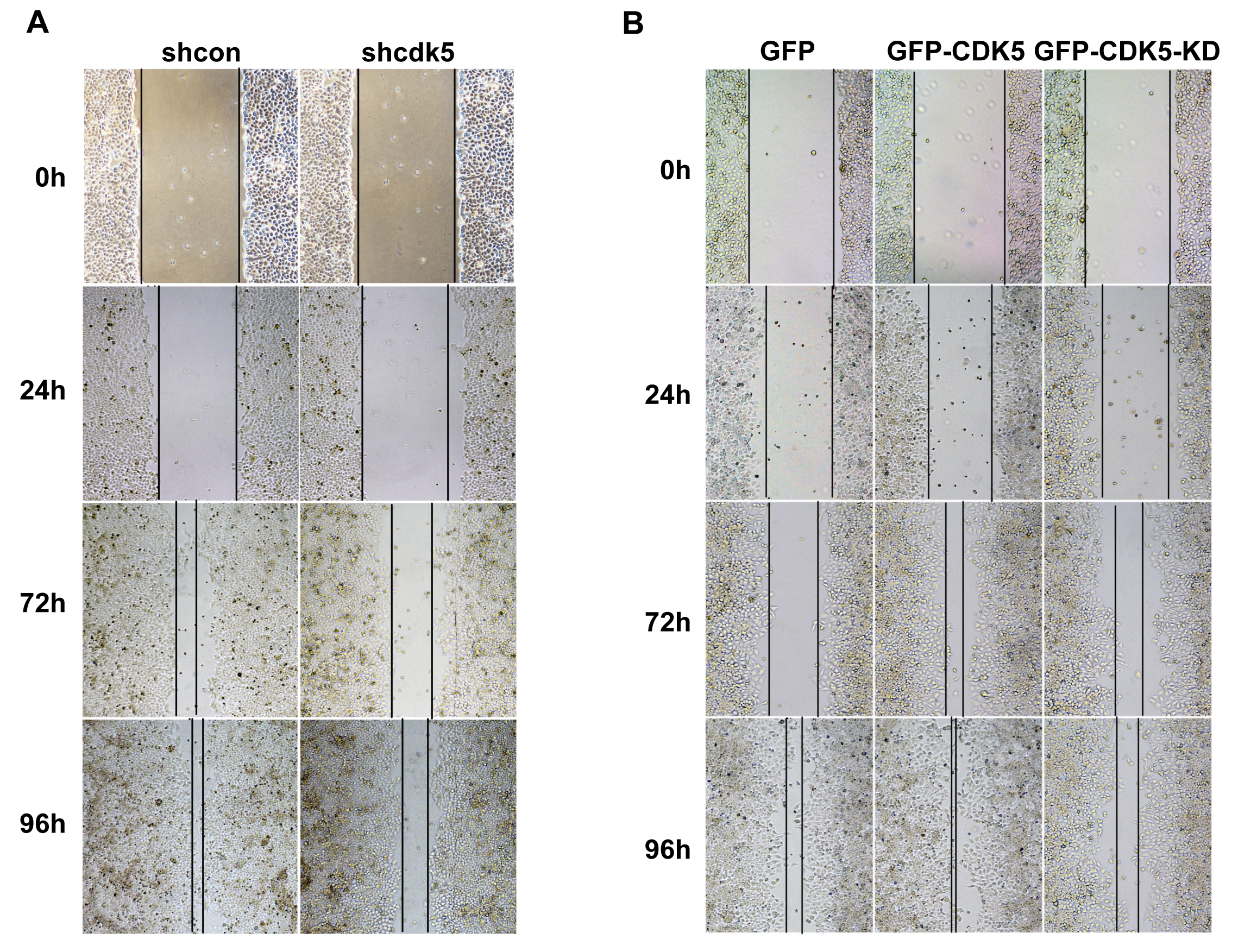

Supplement: Supplementary file 2 — Figure S2. CDK5 is associated with HCC cell migration. a The ability of cell motility was compared in shcon and shcdk5 cells by wound healing assay; Light microscopicimages were taken at 0, 24, 72 and 96 h. b Wound-healing assays were performed in GFP,GFP-Cdk5, GFP-CDK5-KD Huh7 cells; Light microscopicimages were taken at 0, 24, 72 and 96 h. (TIF 8749 kb) [file 13046_2019_1297_MOESM2_ESM.tif]

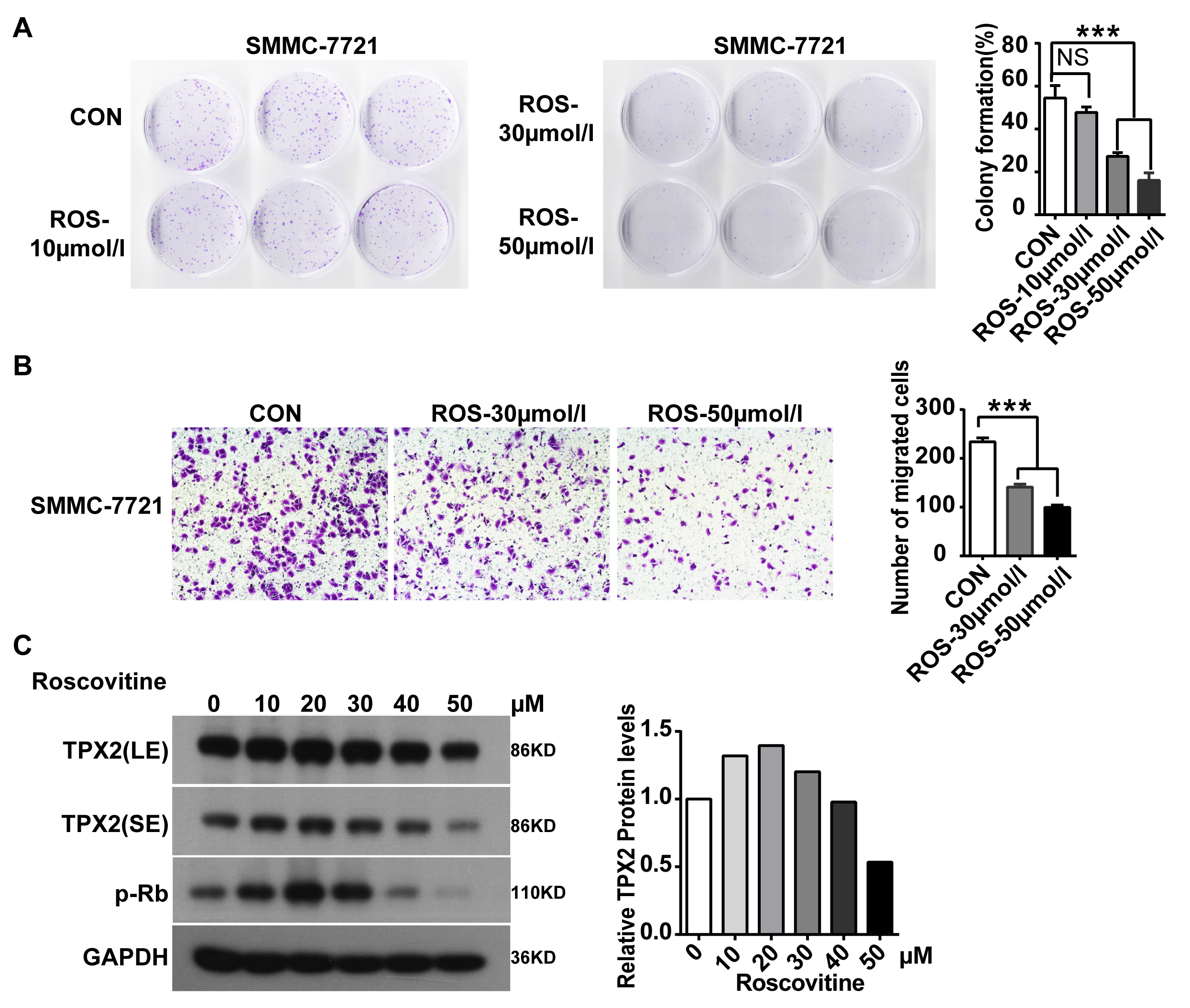

Supplement: Supplementary file 3 — Figure S3. HCC cell proliferation and migration were inhibited by roscovitine. a Colony formation assay inSMMC-7721 cells treated with roscovitine concentration gradient of 10 μmol/l,30 μmol/l,50 μmol/l; One Way ANOVA on Ranks:ns-no statistical differences,***p < 0.001, n = 3. b migration (24 h) assays in SMMC-7721 cells after treatment of roscovitine (30 μmol/l,50 μmol/l);One Way ANOVA on Ranks: ***p < 0.001, n = 5. c Protein levels of TPX2 and p-RB in SMMC-7721 cells after treatment of concentration gradient roscovitine; The average of relative TPX2 protein levels are presented in the right panel. (TIF 15418 kb) [file 13046_2019_1297_MOESM3_ESM.tif]

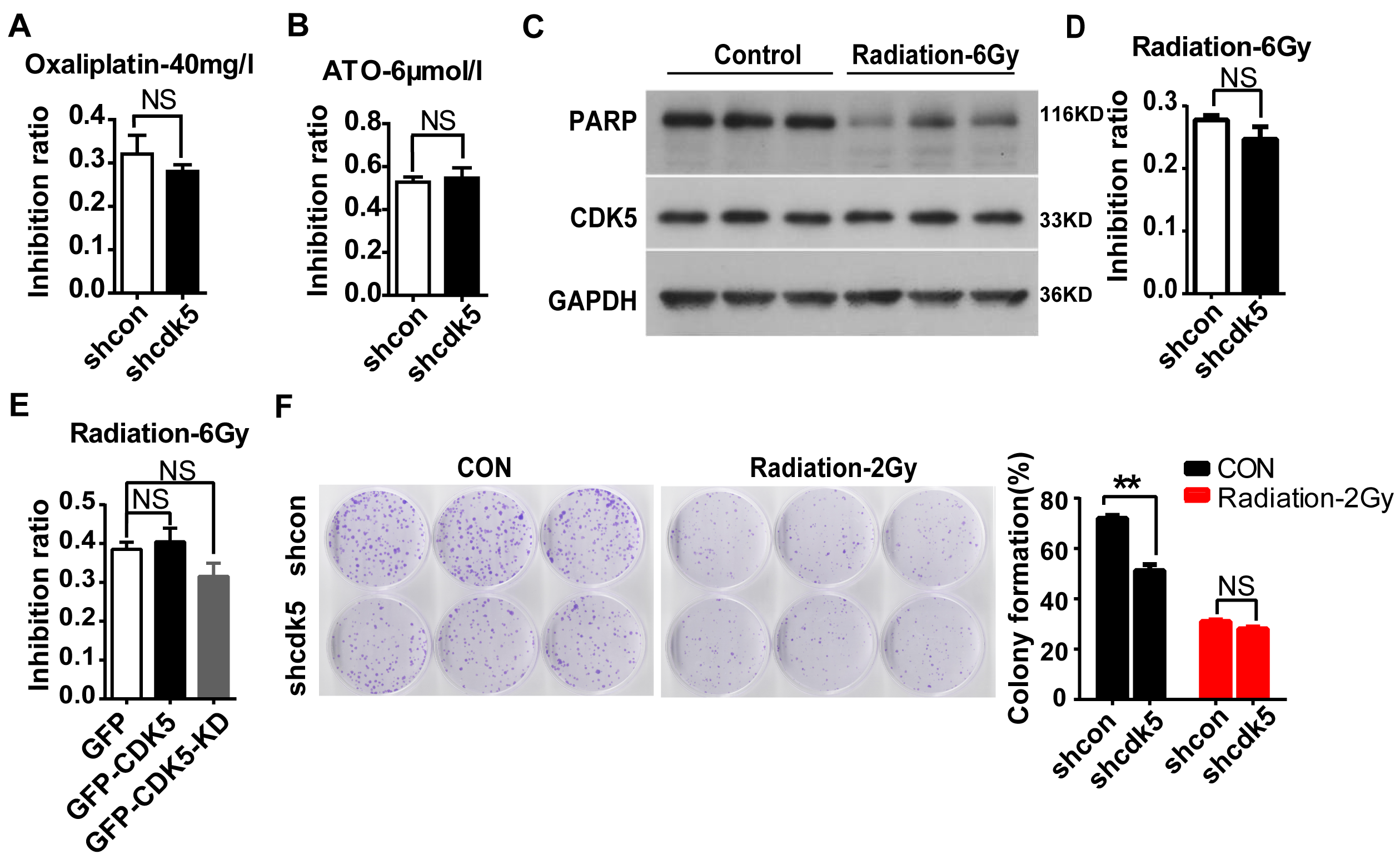

Supplement: Supplementary file 4 — Figure S4. CDK5 has no effect on radiation and chemotherapy induced HCC cell death. a Inhibiton effect of oxaliplatin (40 mg/l) in shcon and shcdk5 SMMC-7721 cells by CCK8 assay. b Inhibiton effect of arsenic trioxide (ATO,6 μmol/l) in shcon and shcdk5 SMMC-7721 cells by CCK8 assay. c PARP and Cdk5 protein levels were measured in SMMC-7721 cells after radiation treatment of 6Gy. d-e CCK8 assays in shcdk5 and Cdk5 over-expressed cells compared with control cells after radiation treatment of 6Gy. f Colony formation assay after radiation treatment in shcon and shcdk5 SMMC-7721 cells. (TIF 11506 kb) [file 13046_2019_1297_MOESM4_ESM.tif]

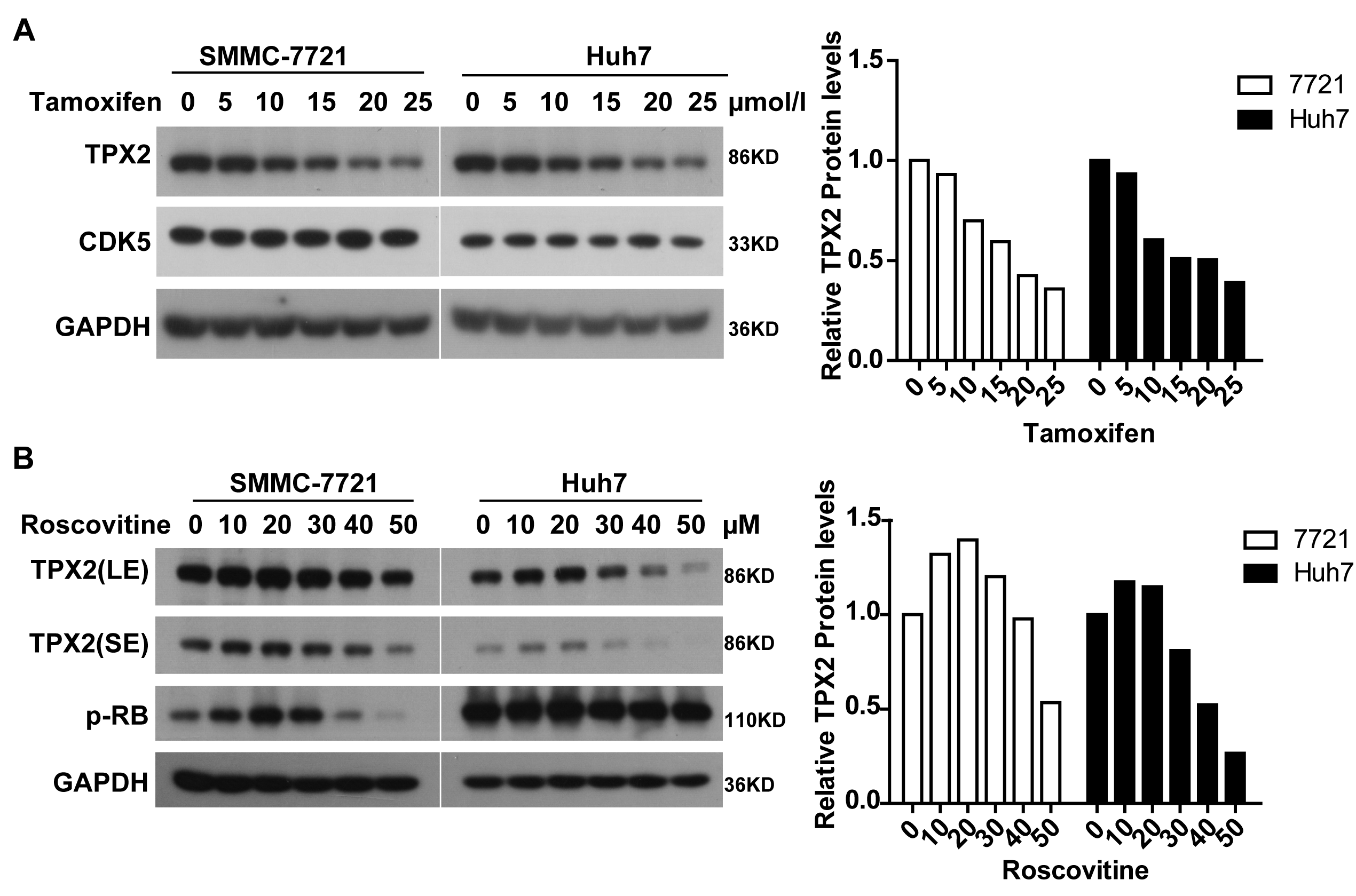

Supplement: Supplementary file 5 — Figure S5. CDK5 inhibitor reduces TPX2 protein level. a Treatment of SMMC-7721 and Huh7 cells with tamoxifen by concentration gradient, TPX2 and Cdk5 protein levels were measured by Immunoblotting,TPX2 protein levels arepresented in the right panel.b Similar to a, SMMC-7721 and Huh7 cells were treated with roscovitine by concentration gradient, TPX2 andp-Rb protein levels were measured by Immunoblotting, TPX2 protein levels arepresented in the right panel. (TIF 12532 kb) [file 13046_2019_1297_MOESM5_ESM.tif]
